# A Double-Blind Randomized Controlled Trial Investigating the Most Efficacious Dose of Botulinum Toxin-A for Sialorrhea Treatment in Asian Adults with Neurological Diseases

**DOI:** 10.3390/toxins7093758

**Published:** 2015-09-22

**Authors:** Mazlina Mazlan, Shivani Rajasegaran, Julia Patrick Engkasan, Ouzreiah Nawawi, Khean-Jin Goh, Saini Jeffery Freddy

**Affiliations:** 1 Department of Rehabilitation Medicine, Faculty of Medicine, University of Malaya, 12th Floor, Menara Selatan, University Malaya Medical Centre, Jalan Universiti, 59100 Kuala Lumpur, Malaysia; E-Mails: drshivani@outlook.com (S.R.); julia@ummc.edu.my (J.P.E.); 2 Department of Biomedical Imaging, Faculty of Medicine, University of Malaya, 12th Floor, Menara Selatan, University Malaya Medical Centre, Jalan Universiti, 59100 Kuala Lumpur, Malaysia; E-Mail: ouzreiah@ummc.edu.my; 3 Neurology Division, Department of Medicine, Faculty of Medicine, University of Malaya, Jalan Universiti, 50603 Kuala Lumpur, Malaysia; E-Mail: gohkj@ummc.edu.my; 4 KPJ KL Rehabilitation Centre, Tawakkal Health Centre, 202A, Jalan Pahang, 53000 Kuala Lumpur, Malaysia; E-Mail: sainijeff@gmail.com

**Keywords:** botulinum toxin type A, neuromuscular agents, sialorrhea, salivary gland diseases, nervous system diseases, stroke

## Abstract

This study aims to determine the most efficacious dose of Botulinum neurotoxin type A (BoNT-A) in reducing sialorrhea in Asian adults with neurological diseases. A prospective, double-blind randomized controlled trial was conducted over 24 weeks. Thirty patients with significant sialorrhea were randomly assigned to receive a BoNT-A (Dysport^®^) injection into the submandibular and the parotid glands bilaterally via an ultrasound guidance. The total dose given per patient was either BoNT-A injection of (i) 50 U; (ii) 100 U; or (iii) 200 U. The primary outcome was the amount of saliva reduction, measured by the differential weight (wet *versus* dry) of intraoral dental gauze at baseline and at 2, 6, 12, and 24 weeks after injection. The secondary outcome was the subjective report of drooling using the Drooling Frequency and Severity Scale (DFS). Saliva reduction was observed in response to all BoNT-A doses in 17 patients who completed the assessments. Although no statistically significant difference among the doses was found, the measured reduction was greater in groups that received higher doses (100 U and 200 U). The group receiving 200 U of Dysport^®^ showed the greatest reduction of saliva until 24 weeks and reported the most significant improvement in the DFS score.

## 1. Introduction

Sialorrhea is a well-recognized disabling symptom associated with 10% of chronic neurological diseases [1,2]. Significant sialorrhea can lead to social and functional impairments, including social embarrassment and isolation, oral and skin fungal infections, aspiration, skin maceration, halitosis, and dehydration [1,2,3,4,5]. Both non-invasive interventions (e.g., oral medications) and invasive interventions (e.g., surgery) are currently offered as treatments for the management of sialorrhea. Among these interventions, botulinum toxin-A injections (BoNT-A) to the salivary glands are considered minimally invasive and have been introduced as a non-permanent treatment for sialorrhea [6]. Based on the current level of evidence, both abobotulinumtoxin A (Dysport^®^, Paris, France) and onabotulinumtoxin A (Botox^®^, Irvine, CA, USA) are considered effective for the treatment of sialorrhea with limited side effects [7].

A previous meta-analysis study has shown the efficacy of BoNT-A compared with placebo in reducing sialorrhea in various neurological diseases such as Parkinson’s disease (PD), amyotrophic lateral sclerosis, and cerebral palsy, but the dose and preparation of BoNT-A has varied across studies [8]. The total dose of Botox^®^ used in those studies ranged from 40 U to 100 U. On the other hand, the total dose of Dysport^®^ used in studies for sialorrhea treatment in adult patients has ranged from 250 U to 450 U [9,10]. Based on a dosing conversion of 1:3 from Botox^®^ to Dysport^®^ [11], the total doses of Dysport^®^ injected were slightly higher. The 2010 International Consensus Statement recommended a Dysport^®^ dose between 15 U to 75 U for each submandibular and parotid gland, resulting in a total minimum dose of 60 U and a total maximum dose of 300 U. However, this consensus statement was based primarily on studies that included both pediatric and adult populations with sialorrhea [12].

To the best of our knowledge, no study has directly compared the efficacy and safety profile of different doses of BoNT-A in adult patients with sialorrhea. The current dosage recommendations are based on research conducted in Western countries; the safety profile may be different among Asian patients. Asian populations are generally physically smaller in size and a previous survey has shown that lower BoNT-A doses were commonly used in Asian countries, especially in BoNT-A intramuscular injections [13]. There are also country-specific recommendations for the total doses of BoNT-A used; found within the summary of product characteristics or prescribing information, especially for Dysport^®^. Thus, we conducted a study comparing different doses of BoNT-A in a cohort of adult neurological patients with sialorrhea to determine the lowest and safest effective dose of BoNT-A in our population.

## 2. Results

### 2.1. Participants

Between September 2010 and February 2014, 41 patients were identified for the study. Eleven patients were excluded (four did not meet the inclusion criteria and seven refused to participate). Thirty patients were eligible and agreed to participate in the study as shown in Table 1 (20 males, 10 females; mean age 56 ± 16.1 years). Of these patients, 19 patients had suffered from a stroke; four patients had a traumatic brain injury; two patients had a motor neuron disease and PD, respectively; and one patient each had encephalitis, cerebral palsy, and a cerebellopontine angle tumor. Out of 19 stroke patients recruited in this study, 12 presented with ischemic strokes and 7 presented with hemorrhagic strokes. Ten stroke patients were on percutaneous endoscopic gastrostomy tube feeding and 12 had cognitive impairment. All patients in the study presented with sialorrhea for more than six months in duration. The patients were randomized to receive 50 U (*n* = 10), 100 U (*n* = 10), or 200 U (*n* = 10) of BoNT-A.

**Table 1:** The demographics of 30 patients recruited at baseline and the doses of botulinum toxin type A (BoNT-A) allocated. Patients No. 1–17 completed all assessments up to 24 weeks and were included in the analysis of outcome measures.

No.	Age (Years)	Sex (F/M)	Diagnosis	Duration of Disease (Years)	BoNT-A Dose Allocated
1	56	M	Stroke	4	50
2	65	F	Stroke	4	50
3	56	M	Stroke	1	50
4	49	M	Stroke	2	50
5	50	F	Stroke	3	100
6	56	M	Stroke	2	100
7	58	F	Stroke	3	100
8	27	M	Stroke	4	100
9	23	M	TBI	2	100
10	39	M	Encephalitis	2	100
11	55	F	Stroke	3	200
12	70	M	Stroke	3	200
13	83	F	Stroke	3	200
14	73	M	Stroke	1	200
15	63	M	Stroke	1	200
16	54	F	TBI	3	200
17	68	M	PD	3	200
18	74	M	Stroke	9	50
19	68	F	Stroke	1	50
20	19	M	CP	3	50
21	25	M	TBI	5	50
22	65	M	MND	1	50
23	55	M	CPA Tumour	1	50
24	79	F	Stroke	1	100
25	56	M	Stroke	1	100
26	33	M	TBI	8	100
27	62	F	MND	3	100
28	79	M	Stroke	3	200
29	62	M	Stroke	1	200
30	64	F	PD	4	200

Note: CP = cerebral palsy; TBI = traumatic brain injury; MND = motor neuron disease; CPA = cerebellopontine angle, PD = Parkinson’s disease.

**Figure 1:**
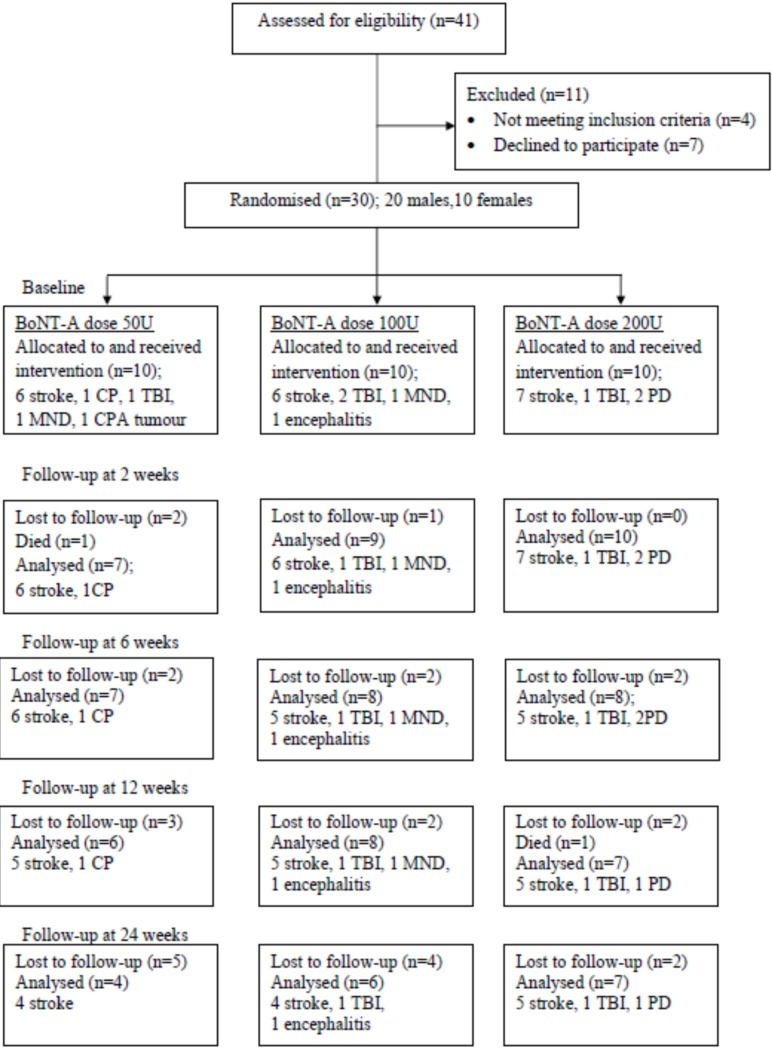
Flowchart describing the recruitment of patients with neurological diseases into the study groups and the dropouts: 17 patients completed all assessments until 24 weeks; four patients in the 50 U group, six patients in the 100 U group and seven patients in the 200 U group. Note: CP = cerebral palsy; TBI = traumatic brain injury; MND = motor neuron disease, CPA = cerebellopontine angle tumour; PD = Parkinson’s disease.

Figure 1 shows the flowchart describing adult patients with neurological diseases participating in the study and the dropouts. There were 10 patients in every group at the start of the study. One patient who received 50 U of BoNT-A died before the first post-injection assessment and therefore, 29 patients were included in the “intent-to-treat” analysis. For the “per-protocol” analysis, only 17 patients completed the follow-up assessments at the end of week 24 and included in the analysis. Two patients who died before the end of the study period and 11 patients who were lost to follow-up were excluded. Patients failed to follow-up because of caregiver’s inability to bring them to the hospital (*n* = 4), progression of neurological disease (*n* = 2), and transportation problems (*n* = 5). One traumatic brain injury patient died from pneumonia two weeks after the injection and one stroke patient died from status epilepticus 12 weeks after enrolment in the study. Both deaths were confirmed to be unrelated to the BoNT-A treatment. No patient dropped out of the study due to treatment side effects. The lowest BoNT-A dose group (50 U) had the highest dropout rate (*n* = 5).

### 2.2. Efficacy Analysis

**Figure 2:**
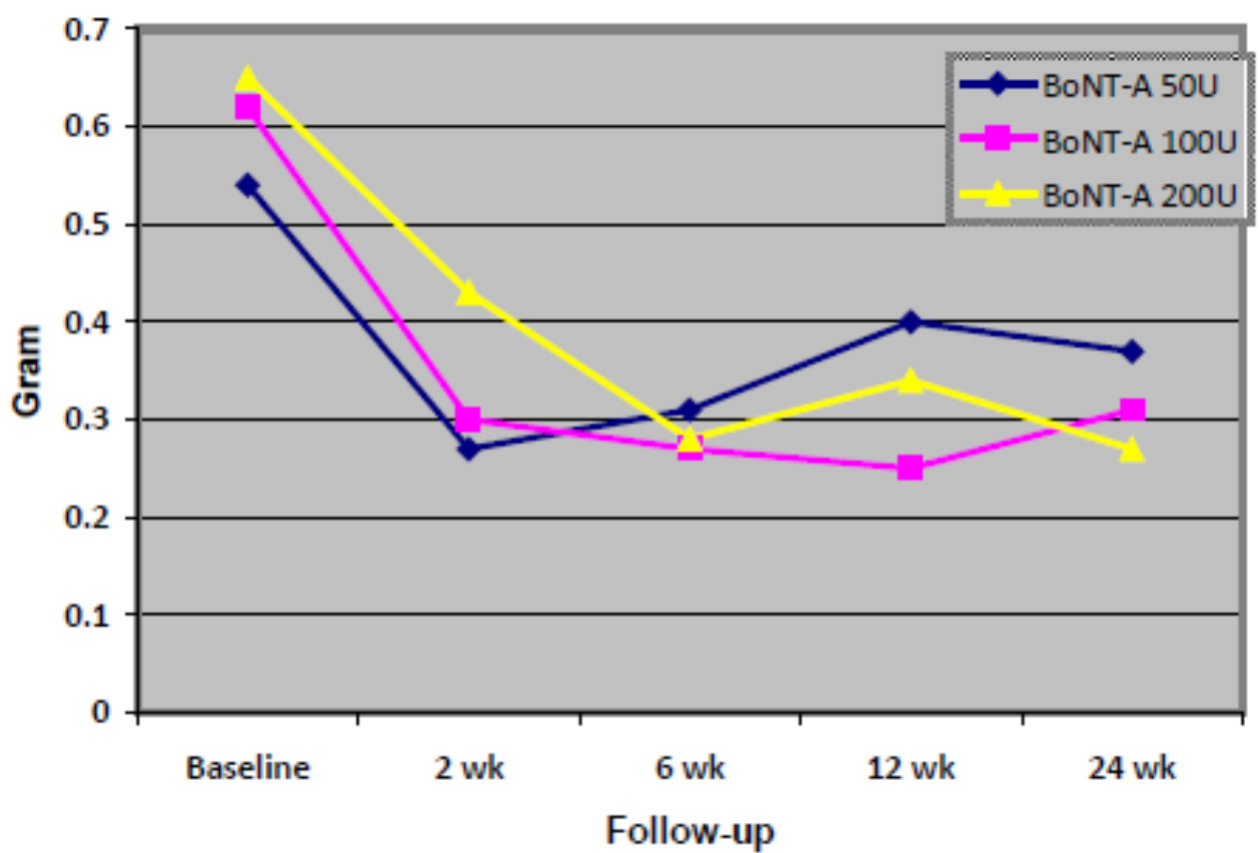
The mean dental gauze weights (wet *versus* dry gauze) in the experimental groups at baseline; before botulinum toxin type A injection, and at 2, 6, 12, and 24 weeks; after botulinum toxin type A injection into bilateral parotid and submandibular salivary glands (*n* = 17). *Note*; BoNT-A = botulinum toxin type A.

The results from the “intent-to-treat” and “per-protocol” analyses are discussed together because similar findings were found in both analyses. Figure 2 shows the comparative mean dental gauze weights for 17 patients who completed the study. At 2 weeks post-injection, sialorrhea was reduced in all three BoNT-A dose groups, as seen from the lower mean values of the differential weight between wet and dry gauze compared with baseline.

**Table 2:** The mean dental gauze weights (wet *versus* dry gauze) in the experimental groups at baseline (g); before botulinum toxin type A injection, and at 2, 6, 12, and 24 weeks; after botulinum toxin type A injection into bilateral parotid and submandibular salivary glands (Per-Protocol Analysis, *n* = 17).

Time	50 Units, *n* = 4		100 Units, *n* = 6		200 Units, *n* = 7		*F* Statistic (d.f.) ^a^	*p*-Value
	Mean, g	(SD)	Mean, g	(SD)	Mean, g	(SD)		
Baseline	0.54	(0.603)	0.62	(0.323)	0.65	(0.824)	0.62 (3)	0.626
2 weeks	0.27	(0.267)	0.30	(0.105)	0.43	(0.591)		
6 weeks	0.31	(0.406)	0.27	(0.165)	0.28	(0.299)		
12 weeks	0.40	(0.439)	0.25	(0.082)	0.34	(0.476)		
24 weeks	0.37	(0.342)	0.31	(0.193)	0.27	(0.271)		

Note: SD = Standard Deviation; d.f. = Degrees of Freedom; ^a^ Repeated measures ANOVA (Time Effect) using Greenhouse-Geisser.

The mean values at baseline and two weeks post-injection for each BoNT-A dose in the “per-protocol” analysis were: 0.54 g *versus* 0.27 g for 50 U, 0.58 g *versus* 0.28 g for 100 U, and 0.53 g *versus* 0.34 g for 200 U (Table 2). In the “intent-to-treat” analysis, the mean values at baseline and two weeks post-injection for each BoNT-A dose were: 0.54 g *versus* 0.27 g for 50 U, 0.58 g *versus* 0.28 g for 100 U, and 0.53 g *versus* 0.34 g for 200 U (Table 3).

**Table 3:** The mean dental gauze weights (wet *versus* dry gauze) in the experimental groups at baseline (g); before botulinum toxin type A injection, and at 2, 6, 12, and 24 weeks; after botulinum toxin type A injection into bilateral parotid and submandibular salivary glands (Intent-to-Treat Analysis, *n* = 29).

Time	50 Units, *n* = 9		100 Units, *n* = 10		200 Units, *n* = 10		*F* Statistic (d.f.) ^a^	*p*-Value
	Mean, g	(SD)	Mean, g	(SD)	Mean, g	(SD)		
Baseline	0.54	(0.419)	0.58	(0.305)	0.53	(0.703)	0.58 (4)	0.687
2 weeks	0.27	(0.185)	0.28	(0.098)	0.34	(0.502)		
6 weeks	0.30	(0.260)	0.33	(0.229)	0.23	(0.257)		
12 weeks	0.33	(0.287)	0.30	(0.237)	0.28	(0.403)		
24 weeks	0.34	(0.231)	0.34	(0.271)	0.22	(0.237)		

Note: SD = Standard Deviation; d.f. = Degrees of Freedom; ^a^ Repeated measures ANOVA (Time Effect) using Greenhouse-Geisser.

Thereafter, the mean dental gauze weight in the BoNT-A 50 U group increased until 24 weeks, which indicates that the efficacy of the injection declined after two weeks. However, the mean dental gauze weight was reduced by 31.5% at 24 weeks compared with baseline. In contrast, the mean dental gauze weight continued to decrease in the BoNT-A 100 U and 200 U groups until 24 weeks post-injection. The lowest mean dental gauze weight was observed in the highest dose (200 U) at the end of the follow-up period. Although a reduction of sialorrhea was observed in both the 100 U and 200 U dose groups from baseline to the end of the follow-up period (by 50% and 58.5%, respectively), the reduction from baseline and between the two groups were not statistically significant (Table 2).

The estimated mean total Drooling Frequency and Severity Scale (DFS) scores at baseline and during each follow-up are reported in Table 4. At two weeks post-injection, a significant reduction in the subjective evaluation of sialorrhea was observed in the group receiving BoNT-A 200 U only, based on the definition of a more than 2-point decrease in the DFS score from baseline. At six weeks post-injection, a significant reduction in the DFS score was observed in both the BoNT-A 100 U and 200 U dose groups, and this reduction persisted throughout the follow-up period.

**Table 4:** Drooling Frequency and Severity Scale (DFS) as described by Thomas-Stonell and Greenberg [14] before and after botulinum toxin type A (BonT-A) injection into bilateral parotid and submandibular salivary glands.

BoNT-A Group (Dysport^®^)	Mean DFS Total Score (Drooling Severity + Frequency)				
	Baseline (SD)	Week 2 (SD)	Week 6 (SD)	Week 12 (SD)	Week 24 (SD)
**50 U**	6.2 (0.66)	4.2 (0.97)	4.1 * (1.16)	4.4 (1.40)	4.7 (1.11)
**100 U**	7.2 (1.39)	5.6 (1.26)	5.1 * (1.19)	4.5 * (0.97)	4.8 * (1.48)
**200 U**	7.5 (1.26)	4.4 * (1.26)	4.7 * (0.70)	4.2 * (1.03)	4.0 * (0.92)

Note: * clinically significant score reduction (based on the definition of more than 2-point decrease in the DFS score from baseline).

### 2.3. Safety

Side effect data were gathered from all contactable patients, including those who dropped out of the study. The only reported toxin-related side effect was the modification of saliva thickness, which led to the transient viscous saliva observed in one patient. The injection-related adverse events were negligible. Two patients reported pain at the injection site during the procedure. None of the treated patients reported xerostomia, dysphagia, dental problems, facial weakness, or aspiration after the BoNT-A injection.

## 3. Discussion

The present study confirmed the effectiveness of BoNT-A in the treatment of sialorrhea for Asian adults with neurological diseases, consistent with the previous findings [6,8,9,10,15]. The maximum BoNT-A dose administered was slightly lower than the common recommendations from previous studies conducted in the West and may be more appropriate for the smaller-sized Asian patient. The doses chosen in this study were conservative and guided by both the literature and the clinical experience of the authors with respect to sialorrhea management. Our findings showed that a total dose of 200 U Dysport^®^ injected into the bilateral submandibular and parotid glands was the most effective in reducing saliva production. Although all three BoNT-A doses (50 U, 100 U, and 200 U) were effective after the first two weeks of injection, differences emerged thereafter with persistent reduction of sialorrhea in 100 U and 200 U groups up to 24 weeks, suggesting greater efficacy with higher doses.

The trend towards a larger sialorrhea reduction with higher doses cannot be ignored despite no statistical differences were observed in the reduction of the differential gauze weight among the three BoNT-A doses in the present study. A meta-analysis examining the effects of BoNT at low and high doses found that both doses produced significant improvements in drooling severity, although the studies using low doses of BoNT were found to have greater outcome heterogeneity than those using high doses [8]. Higher doses of BoNT type B also were found to be more effective in the treatment of sialorrhea among patients with cerebral palsy [16]. In addition, this effect was also observed in patients with various neurological disorders during repeated BoNT injections [17].

In previous studies, the total dose of BoNT-A used ranged from Dysport^®^ equivalent of 50 U to 450 U [8,9,10,18]. Although the potency of Dysport^®^ is different compared to Botox^®^, it is estimated that 100–200 U of Dysport^®^ would correspond to roughly 30–60 U of Botox^®^ [11]. Nevertheless, it was recommended that dosing of BoNT-A should be based on trials conducted with similar BoNT-A formulation rather than an absolute conversion ratio [19]. In addition, direct comparisons between the doses administered in previous studies are not straightforward due to other factors, such as the differences in the number of salivary glands injected and the proportion of BoNT-A injected into the glands.

Injections were performed in both the parotid and the submandibular glands because previous studies have demonstrated the superiority of this combined treatment to achieve the most optimal outcome with a minimal amount of toxin [2,10,12,20]. However, the BoNT-A doses injected into the parotid and submandibular glands vary across studies. In most studies, the dose injected into the parotid glands is higher than that injected into the submandibular glands. For example, Porta *et al*., injected twice the amount of BoNT-A into the parotid compared with the submandibular gland (mean 27.7 U of Botox^®^ per parotid gland *versus* 11.9 U per submandibular gland) in adults with amyotrophic lateral sclerosis [1]. Similarly, Giess *et al*., injected higher doses of BoNT-A at a higher ratio into the parotid gland compared with the submandibular gland (mean 46 U of Botox^®^ per parotid gland *versus* 5 U per submandibular gland) [21]. In our protocol, the BoNT-A dose was divided equally among all the glands regardless of the groups, in accordance with the recommendation from the European International Consensus statement [12].

No side effects or adverse events such as dry mouth (xerostomia), increased saliva thickness or dysphagia, related to BoNT-A were reported in any of the three doses studied, despite injecting all four salivary glands. The injection procedure used an aseptic technique and ultrasound guidance performed by an experienced interventional radiologist, which may have contributed greatly to the precision of the drug penetration and reduced the chance of the toxin spreading to surrounding muscles and damaging the vascular or nerve structures or causing ductal infections. Ultrasound-guided BoNT-A injection into the parotid and submandibular glands is currently regarded as desirable as it is more effective compared to blind technique or using only anatomical landmarks; and with less incidence of side effects from extraglandular BoNT-A infiltration [22,23].

Information from patients and caregivers on drooling severity and frequency reduction is important as it represents a clinically meaningful outcome. In the present study, we note the similarities between the patient’s perception of sialorrhea severity and the actual dental gauze weight measured. Both the groups receiving 100 U and 200 U of BoNT-A reported significant reductions in the severity and frequency of sialorrhea at 24 weeks based on the total DFS scores. However, it is important to note that the group receiving 200 U reported the biggest DFS score improvement from baseline and represented the greatest improvement in the subjective sialorrhea severity. A similar trend was observed with the dental gauze weight. The group injected with 200 U of BoNT-A had the biggest difference in dental gauze weight measurement at 24 weeks compared to baseline, which represented the greatest improvement in the objective sialorrhea severity.

In this study, the relatively high dropout rate was unavoidable despite extra measures taken to improve follow-up compliance such as assisting with transportation and frequent telephone reminders. The reasons that patients were lost to the follow-up were related primarily to issues with immobility and transportation. The group that received the lowest dose (50 U) had the highest dropout rate. Since majority of the dropouts in that group occurred at the later visits, especially on the last visit at 24 weeks, the reduced efficacy of the lowest dose cannot be ruled out as a reason for the relatively high dropout rate.

Previous prospective, longitudinal studies using BoNT with repeated saliva measurements have also suffered from high dropout rates. The dropout rate was between 40% to 50% [9,16,24] which is similar to the present study. Reasons for the dropouts were not specified and the highest dropouts were not necessarily from the lowest BoNT-A dose. Although the dropout rate may reduce the overall statistical strength of this study, we believe that the finding can still assist physicians to optimize sialorrhea treatment. Furthermore, the total number of patients included in the final analysis in most randomized controlled trials using different BoNT doses ranged from 12 to 25; fitting with the 17 patients analyzed in the present study [7,9,24,25].

Patient recruitment took almost four years to complete, as this procedure is not routinely offered in this country and patients preferred other non-invasive treatments for their sialorrhea. In addition to being unfamiliar with the procedure, most participants with significant sialorrhea were in the late or severe stages of their disease, and therefore these patients were dependent on their caregivers to attend the frequent follow-up visits. Difficulties in complying with the subsequent follow-up visits resulted in almost half of the patients dropping out of the study before the end of the follow-up period. Therefore the results of this study should be interpreted with these limitations in mind.

Ideally, this study should be performed on the basis of a specific neurological diagnosis and on a larger sample size. Previous studies and reviews in sialorrhea predominantly concerned patients suffering from neurodegenerative disorders, notably in PD [7,9,17,26], whereas in the present study, two-thirds of the patients recruited suffered from stroke. The main reason was due to the high number of stroke patients admitted in Neurorehabilitation, even after all adult patients with neurological diseases and sialorrhea were screened. However, the positive results from BoNT-A injection indicate that stroke patients also benefit from this treatment.

In conclusion, all investigated doses of BoNT-A (Dysport^®^) injected into the bilateral parotid and submandibular salivary glands were effective and safe in treating sialorrhea in Asian adult patients. The higher dose of 200 U of BoNT-A was more efficacious than the other two doses to reduce sialorrhea for up to 24 weeks, although this dosage was lower than commonly recommended dose for sialorrhea treatment in most studies.

## 4. Experimental Section

### 4.1. Design and Procedure

This study was designed as a prospective, randomized, double-blind trial evaluating the efficacy and safety of three different doses of BoNT-A injected into the bilateral parotid and submandibular glands. All patients who were aged 18 years or above, had a neurological disease and significant sialorrhea, and were seen at the Neurorehabilitation Clinic at the University of Malaya Medical Centre (UMMC) in Kuala Lumpur, were screened and recruited for the study. All patients or their legal guardians provided informed consent. Sialorrhea was deemed to be significant if it was reported to be socially inappropriate or causing discomfort, as defined by a score of five or more according to the DFS [14]. Patients who were on anticholinergic drugs (e.g., patients with Parkinson’s disease) were included in the study; however, it was required that their anticholinergic medication dose remain unchanged throughout the study period. Pregnant participants and those on anticoagulant medications were excluded from the study.

Eligible patients were assigned to receive one of the three BoNT-A doses (50 U, 100 U, or 200 U) according to a pre-determined randomization schedule generated using the SE Strata Version 9.0 block randomization program. The randomization list was labelled A, B and C to indicate the three BoNT-A doses and then arranged in standard sealed envelopes labelled 1 to 30 by the study coordinator. The study coordinator kept the master list confidential and secure and assigned the envelope to patients who enrolled in the study in a sequential manner.

BoNT-A (Dysport^®^ 500 U toxin-hemagglutinin complex, human albumin and lactose, freeze-dried powder for injection) vials were kept in a refrigerator at 2 °C. On the day of the injection, the vials were prepared by a research assistant in a separate lab and delivered to the clinic, which was located within the same vicinity, for immediate injection. The patients, their caregivers, and the clinicians involved in the study were blinded to the dose of BoNT-A injected throughout the study period. Blinding was maintained until all the patients had completed the study, and data collection was complete or when a serious adverse event occurred. 

Dilution of the drug was carried out according to the two-step procedure used by Mancini F *et al*. [10]. All the vials were initially reconstituted with 1 mL of 0.9% normal saline to achieve a mother solution of 500U/mL. Another 1 mL syringe was prepared to obtain different diluted solutions: (i) for 50U of BoNT-A; 0.1 mL was drawn from the mother solution and 0.9 mL of 0.9% saline was added (ii) for 100U of BoNT-A; 0.2 mL was drawn from the mother solution and 0.8 mL of 0.9% saline was added (iii) for 200U of BoNT-A, 0.4 mL was drawn from the mother solution and 0.6 mL of 0.9% saline was added. The dilutions were standardized to 1 mL for all three doses.

Prior to the injection, the vascular anatomy of the parotid and submandibular glands and the intraparotid tract of the patient’s facial nerve were briefly assessed by a radiologist (one of the co-investigators) via an ultrasound. BoNT-A then was injected into the middle aspect of the submandibular glands and the superficial lobe of parotid glands bilaterally using a 1-mL syringe and a 25- or 23-gauge needle depending on the gland size. The injection was given as a single shot centrally within the gland. Each gland was injected with 0.25 mL solution providing an equal amount of toxin. The total dose received per patient was 50 U, 100 U, or 200 U. No injection was made into the deep lobe of the parotid glands. The injection was performed under ultrasound guidance without anesthesia using a strict aseptic technique.

All patients were assessed five times throughout the study period. Baseline assessments were conducted just prior to the injection. Subsequent assessments were conducted 2, 6, 12, and 24 weeks post-injection. The primary outcome measure was the objective amount of saliva produced, which was measured as the difference in weight of a dry and wet standardized dental gauze at each assessment. The authors used a two-ply standard dental gauze that was rolled into a cylindrical shape with the exact measurements of 2 cm in length and 0.5 cm in diameter. The dry weight was measured before placement and the wet weight was measured after placement in the participant’s buccal mucosal cavity for five minutes. An average of three measurements was taken at an interval of one minute. Differences in weight were calculated via an electronic microbalance scale to the nearest 0.0001 g. Patients were assessed at approximately the same time of day at each visit and were advised not to take anything orally or by gastrostomy tube for at least one hour prior to the assessment.

The secondary outcome measure was the subjective report of saliva production by the patients or their caregivers using the DFS [14]. The DFS was rated using two subscales: (1) drooling severity, with a scale from 1 to 5 where 1 = never drools, 2 = mild drooling causing wet lips only, 3 = moderate drooling causing wet lips and chin, 4 = severe drooling where clothing becomes damp, and 5 = profuse drooling causing clothing, hands, and the patients in general to get wet; and (2) drooling frequency, with a scale from 1 to 4 where 1 = never drools, 2 = occasionally drools, 3 = frequently drools, and 4 = constantly drools. The total score was determined by adding the two subscales together (drooling severity + drooling frequency). The DFS was rated from 2 to 9, with a ranking score of 2 representing no drooling and a ranking score of 9 representing the most severe level of drooling. We defined a more than 2-point decrease in the DFS as successful treatment.

Any adverse effects and complications were recorded and carefully documented throughout the study. Adverse events were assessed by questioning the patients and their caregivers at each study visit. The relationship of an adverse event to the study drug was classified as probable, possible, not related, or not assessable by the investigator.

This study was designed originally to be a pilot study with an expected high rate of dropouts, thus we did not attempt to calculate the sample size necessary to achieve statistical significance. Nevertheless, a study powered to detect a 20% difference in our primary outcome measure (dental gauze weight) with 80% sensitivity was initially attempted. Ethical approval was obtained from UMMC Ethics Committee and this study is registered at ClinicalTrials.gov, (registration number NCT02425176).

### 4.2. Statistical Analysis

Statistical analysis was performed using the Statistical Package for the Social Sciences software version 17.0 (SPSS 17.0). Repeated measures analysis of variance (ANOVA) tests were used to assess differences in the mean dental roll gauze weight among the three dose groups followed by a post-hoc test (Bonferroni Test) for any significant results. The level of significance was set at *p* < 0.05. Data normality was tested by means of the Shapiro-Wilk W-test, and non-parametric statistics were used for variables that were not normally distributed.
